# Maternal obesogenic diet enhances cholestatic liver disease in offspring

**DOI:** 10.1016/j.jlr.2022.100205

**Published:** 2022-03-25

**Authors:** Michael D. Thompson, Holly Hinrichs, Austin Faerber, Phillip I. Tarr, Nicholas O. Davidson

**Affiliations:** 1 Division of Endocrinology and Diabetes, Department of Pediatrics, Washington University School of Medicine, St. Louis, MO, USA; 2 Division of Gastroenterology, Hepatology, and Nutrition, Department of Pediatrics, Washington University School of Medicine, St. Louis, MO, USA; 3 Division of Gastroenterology, Department of Internal Medicine, Washington University School of Medicine, St. Louis, MO, USA

**Keywords:** bile acid metabolism, obesity, microbiome, liver, animal models, maternal high fat/high sucrose, cholestatic liver disease, ductular reaction, cecal transplant, NAFLD, ALP, alkaline phosphatase, ALT, alanine transaminase, AST, aspartate transaminase, BA, bile acid, CMT, cecal microbiome transplantation, CON, conventional chow, DDC, 3,5-diethoxycarbonyl-1,4-dihydrocollidine, HF/HS, high fat/high sucrose, HFD, high-fat diet, IBD, inflammatory bowel disease, LW/BW, liver to body weight, MMP, matrix metalloproteinases, NAFLD, nonalcoholic fatty liver disease, PSC, primary sclerosing cholangitis, PSR, picrosirius red

## Abstract

Human and animal model data show that maternal obesity promotes nonalcoholic fatty liver disease in offspring and alters bile acid (BA) homeostasis. Here we investigated whether offspring exposed to maternal obesogenic diets exhibited greater cholestatic injury. We fed female C57Bl6 mice conventional chow (CON) or high fat/high sucrose (HF/HS) diet and then bred them with lean males. Offspring were fed 3,5-diethoxycarbonyl-1,4-dihydrocollidine (DDC) for 2 weeks to induce cholestasis, and a subgroup was then fed CON for an additional 10 days. Additionally, to evaluate the role of the gut microbiome, we fed antibiotic-treated mice cecal contents from CON or HF/HS offspring, followed by DDC for 2 weeks. We found that HF/HS offspring fed DDC exhibited increased fine branching of the bile duct (ductular reaction) and fibrosis but did not differ in BA pool size or intrahepatic BA profile compared to offspring of mice fed CON. We also found that after 10 days recovery, HF/HS offspring exhibited sustained ductular reaction and periportal fibrosis, while lesions in CON offspring were resolved. In addition, cecal microbiome transplant from HF/HS offspring donors worsened ductular reaction, inflammation, and fibrosis in mice fed DDC. Finally, transfer of the microbiome from HF/HS offspring replicated the cholestatic liver injury phenotype. Taken together, we conclude that maternal HF/HS diet predisposes offspring to increased cholestatic injury after DDC feeding and delays recovery after returning to CON diets. These findings highlight the impact of maternal obesogenic diet on hepatobiliary injury and repair pathways during experimental cholestasis.

Current understanding of developmental origins of health and disease teaches that in utero and/or early life exposures influence susceptibility to chronic diseases in later life (1). This concept is relevant to obesity; approximately 55% of women are overweight or obese at conception (2), and maternal obesity is associated with development of metabolic syndrome and its complications in offspring (1, 3, 4, 5), and there is an association between maternal pre-pregnancy body mass index and offspring nonalcoholic fatty liver disease (NAFLD) (6, 7, 8). Rodent and nonhuman primate models demonstrate that exposure to a maternal high-fat diet (HFD) in utero and postnatally (by nursing) increase propensity to develop NAFLD (9, 10, 11, 12). Most data focus on hepatocellular injury and adipose biology, while we know little about the role(s) of maternal obesity and/or obesogenic diets on cholestatic liver disease in offspring.

Cholestasis is characterized by altered bile acid (BA) homeostasis and periportal injury, including inflammation and fibrosis. One such condition is primary sclerosing cholangitis (PSC), which is associated with inflammatory bowel disease (IBD) in 70% of cases (13). The etiology of PSC is ill-defined, and therapies for PSC are limited (14). While formal links have yet to be established between PSC and early life exposure to maternal obesity, animal data show that maternal obesogenic diet increases susceptibility to IBD in offspring (15, 16).

We have shown that BA metabolism is altered in offspring exposed to maternal high fat/high sucrose (HF/HS) diet (17), with increased size of the BA pool and a shift in intrahepatic BA species. Offspring exposed to maternal HF/HS also exhibit mild periportal inflammation and fibrosis, even when fed a low fat, chow diet (17). Here we hypothesize that offspring exposed to maternal obesogenic diets are more sensitive to cholestatic injury. To test this hypothesis, we evaluated the effect of maternal obesity on the development and regression of cholestatic injury in offspring.

## Materials and methods

### Mouse breeding, feeding paradigm, and sample collection (Fig. 1)

All procedures in this study were approved by the Animal Studies Committee at Washington University School of Medicine and conformed to National Institutes of Health guidelines and reporting remained consistent with ARRIVE guidelines. We fed 4-week-old female C57Bl/6J mice either a HF/HS (Test Diet 58R3; 59% fat, 26% carbohydrates [17% sucrose] and 15% protein) or standard chow (CON) (Pico Lab Rodent diet 20; 13% fat, 62% carbohydrates [3.2% sucrose] and 25% protein) for 6 weeks (18). CON- and HF/HS-fed F0 female mice were mated with chow-fed male mice to produce offspring that were weaned onto chow diet. All pups remained with their respective dam until weaning. At 10 weeks of age, mice from each group were fed diet supplemented with 0.1% of 3,5-diethoxycarbonyl-1,4-dihydrocollidine (DDC) for 2 weeks. An additional cohort of mice were transitioned back to chow diet after 2 weeks on DDC to permit recovery from liver injury. Serum and relevant tissues were collected at sacrifice. For these studies, at least five litters were represented in each group. Body and liver weights were measured for all offspring at the time of tissue collection as well as weekly body weights during DDC feeding. For all breeding, potential dams were staged to identify the most likely period for successful mating. The sire was placed in the cage for only 24-h to limit co-housing effects.

**Fig. 1:**
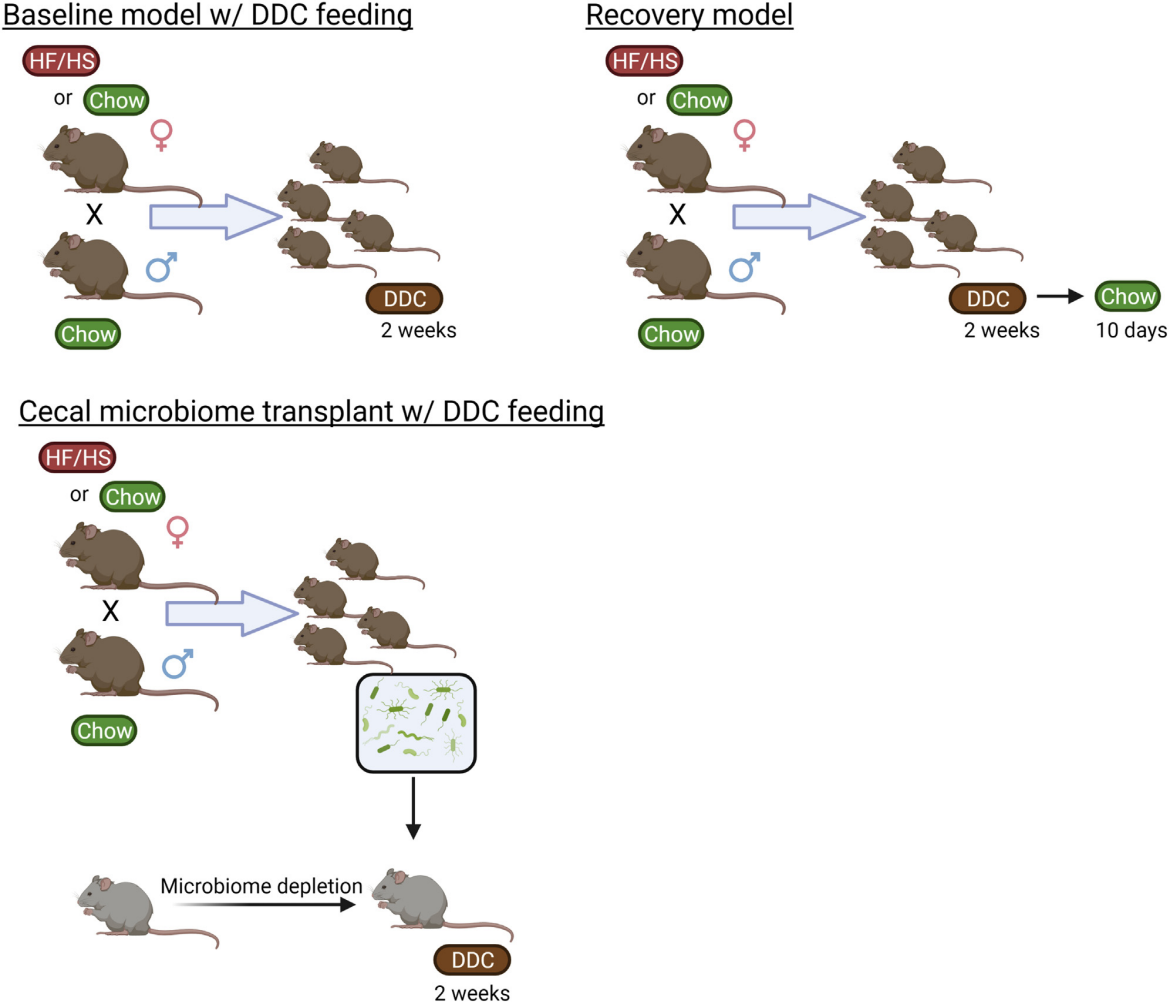
Experimental design. Breeding scheme, diet feeding, and cecal microbiome transplant for mouse models in this manuscript. DDC, 3,5-diethoxycarbonyl-1,4-dihydrocollidine; HF/HS, high fat/high sucrose. Created with BioRender.com.

### Serum analysis

Blood was collected to complete serum analysis. Measurements of alanine transaminase, aspartate transaminase (AST), alkaline phosphatase, and bilirubin were undertaken by the Division of Comparative Medicine Research Animal Diagnostic Laboratory. Serum BAs were measured utilizing the BA assay kit (Crystal Chem, Elk Grove Village, IL).

### Histology, stains, and immunohistochemistry

Tissues fixed in 10% formalin were embedded in paraffin, and 5-μm sections were stained with hematoxylin and eosin and picrosirius red (PSR) as we have reported (19). For immunohistochemistry, sections were rehydrated by passing through xylene, graded alcohol, and distilled water and then stained with select primary antibodies (CD45, Mac-2, and CK-19) and the Vectastain Elite ABC kit (Vector Laboratories, Burlingame, CA) protocol. Antigen retrieval was performed by heating for 15 min in citrate buffer in a pressure cooker, after which we inactivated tissue peroxide, and incubated them with primary antibody (∼20 h, 4°C). Sections were then washed and incubated with biotin-conjugated secondary antibody (30 min) (Vector Laboratories), washed and incubated with ABC reagent, and washed and incubated with 3,3′-diaminobenzidine or alkaline phosphatase reagent. Sections were counterstained with hematoxylin and cover slipped using Cytoseal XYL (Richard Allen Scientific, Kalamazoo, MI). PSR staining, CD45 IHC, and Mac-2 IHC was quantified using ImageJ to calculate the percent area of the section that was positive. Three representative images were taken (with 10× objective) for each mouse with one image coming from each of three different liver lobes. For CK-19, the number of positive ducts were counted per field (20× objective centered on 1 periportal area) with five fields counted across three liver lobes for each animal. For all quantitative graphs for staining, each data point represents a single mouse.

### BA analysis

BA pool size was measured in offspring as previously reported (20). Briefly, after 4 h of fasting gallbladder, liver, and intestines, including luminal contents were collected, weighed, homogenized, and incubated in ethanol to extract BAs. Total BA content was determined enzymatically (Genway Biotech, San Diego, CA) and normalized to body weight. Individual intrahepatic BA species were measured using high-performance liquid chromatography and tandem mass spectrometry as previously reported (20). Mass spectrometry was performed in the Metabolomics Facility at Washington University (P30 DK020579). Individual BA concentrations were normalized to tissue weight and converted to molar concentration. The concentrations of individual hepatic BA types are presented as a percent of the total intrahepatic BA pool. Hydrophobicity index was calculated as previously reported (21).

To evaluate Cyp7a1 activity, serum concentrations of 7α-hydroxy-4-cholesten-3-one (C-4) were measured using protein precipitation to extract C-4 from 50 μl of mouse serum in the presence of deuterated internal standards (d7-C-4) (17). C-4 on a SecurityGuard Gemini C18 (4 × 3 mm) and ACE C8 column (3 μm, 100 × 4.6 mm) were detected with positive multiple-reaction monitoring on an Applied Biosystems Sciex 4000QTRAP tandem mass spectrometer.

### Quantitative PCR analysis

Total hepatic RNA was extracted, and cDNA prepared using an ABI high-capacity cDNA reverse transcription kit with 1 μg of total RNA. Real-time quantitative PCR used cDNAs from at least six animals per group and was performed in duplicate on an ABI 7500 sequence detection system using SYBR Green PCR Master Mix (Applied Biosystems) and appropriate primer pairs (provided on request). Relative mRNA abundance is expressed as fold change to CON group after normalization to GAPDH.

### Matrix metalloproteinases activity assay

Total matrix metalloproteinases (MMP) activity was measured in liver extracts utilizing an MMP activity assay kit (Abcam).

### 16S sequencing for gut microbiome analysis

Cecal stool samples were collected and sent to MRDNA (http://www.mrdnalab.com) for 16S rRNA gene sequencing and bioinformatics analysis. Stool DNA was isolated from cecal contents using the PowerSoil® DNA Isolation Kit (Qiagen) per the manufacturer’s instructions. The samples were lysed with beads and spin filtered to elute purified DNA, which was stored at −20°C until PCR amplification. A reengineered version of bTEFAP®, a form of amplicon sequencing utilizing next generation sequencing, was used to evaluate the microbiota. The 16s rRNA primer pair, 515F GTGYCAGCMGCCGCGGTAA/ 806R GGACTACNVGGGTWTCTAAT, was utilized to evaluate the microbial ecology of each sample on the Illumina NovaSeq with methods via the bTEFAP® DNA analysis service. Each sample underwent a single-step 35 cycle PCR using HotStarTaq Plus Master Mix Kit (Qiagen, Valencia, CA) were used under the following conditions: 95°C for 5 min, followed by 35 cycles of 95°C for 30 s; 53°C for 40 s, and 72°C for 1 min; after which a final elongation step at 72°C for 10 min was performed. The amplification products from the different samples were mixed in equal concentrations, purified, and sequenced (Illumina NovaSeq) sequencing.

The Q25 sequence data derived from the sequencing process were processed using the MR DNA ribosomal and functional gene analysis pipeline (www.mrdnalab.com , MR DNA, Shallowater, TX). Sequences are depleted of primers, short sequences <150 bp are removed, and sequences with ambiguous base calls removed. Sequences are quality filtered using a maximum expected error threshold of 1.0 and dereplicated. The dereplicated or unique sequences are denoised; unique sequences identified with sequencing or PCR point errors are removed, followed by chimera removal, thereby providing a denoised sequence or zOTU. Final zOTUs were taxonomically classified using BLASTn against a curated database derived from NCBI (www.ncbi.nlm.nih.gov) and compiled into each taxonomic level into both “counts” and “percentage” files.

Alpha and beta diversity analyses were viewed through Qiime 2. Taxonomic data are presented as abundance relative to all counts.

### Microbiome depletion and cecal microbiome transplantation

Male C57Bl6 mice were purchased from Jackson laboratories (Cat#000664) at 6 weeks old and acclimated to our mouse facility for 1 week before antibiotic treatment (to deplete the resident gut bacterial microbiome). The ABX mix contained 300 ml of sterile water to which vancomycin (150 mg, Chem-Impex, Wood Dale, IL), neomycin (300 mg, Sigma, St. Louis, MO), and artificial sweetener (2 g, Equal, New Providence, NJ) were added. The antibiotic water was provided for 60 h followed by 12 h of water containing polyethylene glycol.

Cecal contents were collected from 6-week-old male CON and HF/HS lineages and placed in PBS diluted with glycerol (adjusted to 16%) to create a slurry at 0.1 g/ml concentration and stored (−80°C) until use. The slurry mix was thawed on wet ice, homogenized with a glass bulb, passed through a 100-μm nylon cell strainer, centrifuged for 3,000 rpm, 30 s, and vortexed to create a homogeneous mix 30 min prior to oral gavage of the recipient mice. Microbiome depleted mice were gavaged with stool slurry from CON or HF/HS offspring five times over 11 days.

### Statistical analysis

Two-way ANOVA with posthoc comparison or unpaired Student’s *t*-tests was used when appropriate using GraphPad prism software and noted in the figure legend. Data are presented as means (±SD) with two-tailed *P* < 0.05 representing significance and two-tailed *P* > 0.05 and <0.10 representing a nonstatistically significant trend. All authors had access to the all data and have reviewed and approved the final manuscript.

## Results

### Offspring exposed to maternal obesogenic diet have increased ductular reaction during DDC feeding

**Fig. 2:**
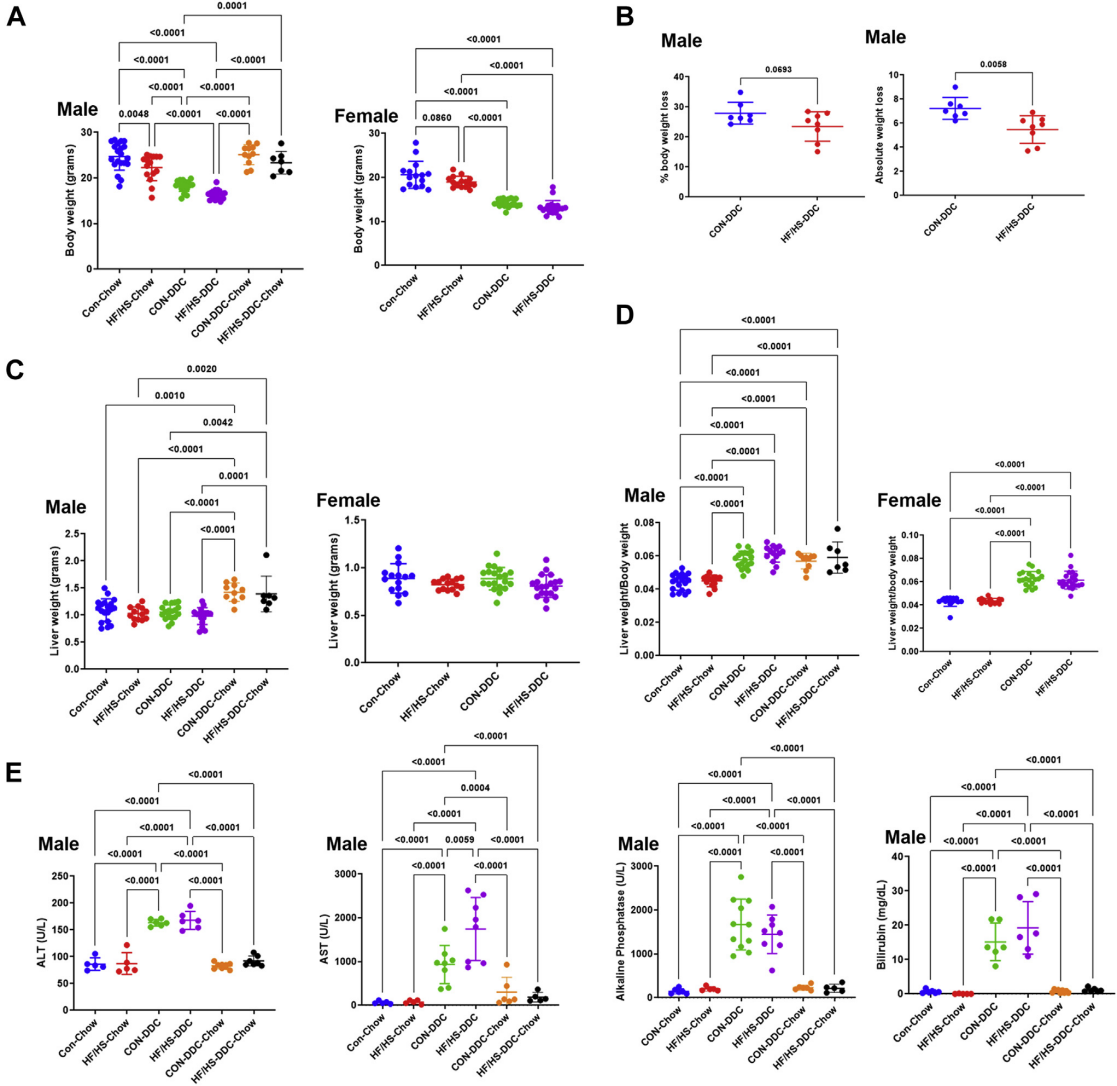
Morphometric and serum analysis in offspring after DDC feeding and recovery. A: Body weights (BW) of male and female offspring from maternal CON and HF/HS lineage fed chow or DDC. Male recovery mice also included. B: Percent and absolute body weight loss in male offspring fed DDC diet for 2 weeks. C: Liver weights (LWs) of male and female offspring from maternal CON and HF/HS lineage fed chow or DDC. Male recovery mice also included. D: Liver to body weight (LW/BW) ratios of male and female offspring from maternal CON and HF/HS lineage fed chow or DDC. Male recovery mice also included. E: Serum analysis for ALT, AST, ALP, and bilirubin in male offspring fed chow, DDC, or DDC followed by chow. ALP, alkaline phosphatase; ALT, alanine transaminase; AST, aspartate transaminase; DDC, 3,5-diethoxycarbonyl-1,4-dihydrocollidine; HF/HS, high fat/high sucrose.

To evaluate the impact of maternal obesogenic diet on offspring cholestatic liver disease, F0 female mice were placed on control chow or HF/HS diet for 6 weeks and mated as previously reported (18). Offspring were fed DDC for 2 weeks to induce cholestatic liver injury (Fig. 1). A separate cohort of mice were transitioned back to regular chow for an additional 10 days to assess recovery from cholestatic injury. Body weight in male and female offspring after 2 weeks of DDC feeding was not significantly different (Fig. 2A). However, absolute weight loss in male offspring was significantly less in HF/HS offspring fed DDC with a similar trend in percent body weight loss (Fig. 2B). No difference in liver weight and liver weight to body weight ratio was present between CON and HF/HS offspring after 2 weeks of DDC feeding (Fig. 2C, D). Serum analysis demonstrated that alanine transaminase, AST, alkaline phosphatase, and total bilirubin all increased after 2 weeks of DDC in CON and HF/HS offspring (Fig. 2E). However, only serum AST showed a significant increase in HF/HS offspring versus CON offspring after 2 weeks of DDC (Fig. 2E).

**Fig. 3:**
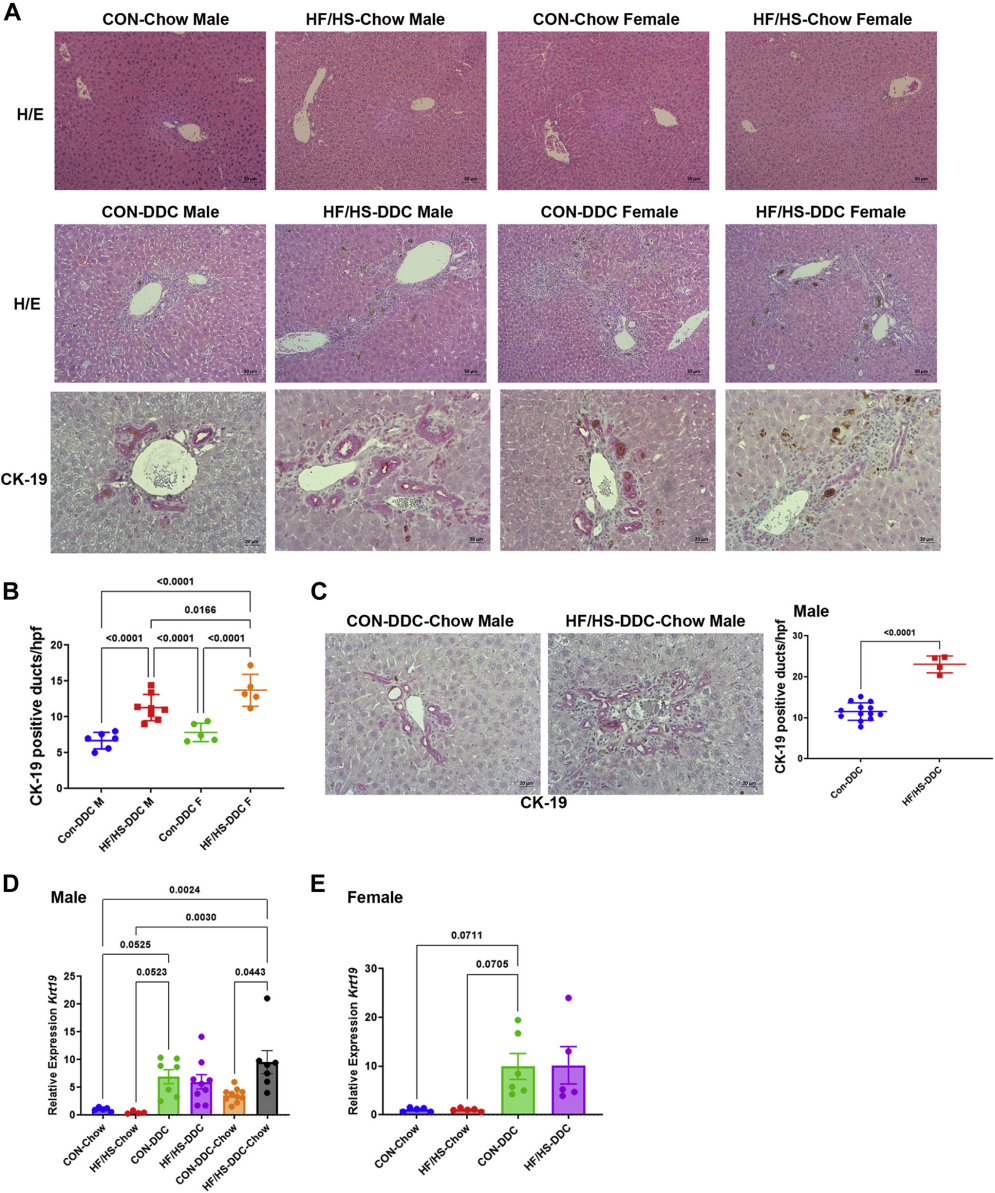
Maternal obesogenic diet increases biliary ductular reaction in offspring fed DDC. A: Representative photomicrographs of H&E staining of liver from male and female offspring fed chow and H&E staining and IHC for CK-19 of liver from male and female offspring fed DDC. B: Quantification of CK-19-positive bile ducts in liver of offspring fed DDC. C: Representative photomicrographs of IHC for CK-19 of liver from male offspring fed DDC for 2 weeks and transitioned to chow for 10 days to recover with quantification of CK-19-positive bile ducts to the right. D: relative expression of *Krt19* in male offspring at baseline, after 2 weeks of DDC, and 2 weeks of DDC with 10 days of chow diet for recovery. E: Relative expression of *Krt19* in female offspring at baseline and after 2 weeks of DDC. Quantitative data presented as mean (±SD) with n ≥ 5 in each group and ≥5 separate litters represented in each group. *P* values indicated on graph. DDC, 3,5-diethoxycarbonyl-1,4-dihydrocollidine; H&E, hematoxylin and eosin; HF/HS, high fat/high sucrose.

DDC feeding induces ductular proliferation due to cholestasis, apparent on hematoxylin and eosin staining (Fig. 3A). CK-19 staining was performed to evaluate the degree of ductular reaction (Fig. 3A). Both male and female offspring from the maternal HF/HS lineage had increased CK-19-positive bile ducts compared to CON (Fig. 3A, B). Gene expression of *Krt19* increased after DDC feeding but was not significantly different between CON and HF/HS offspring (Fig. 3D, E)

### Offspring exposed to maternal obesogenic diet exhibit less inflammation but worse fibrosis after DDC feeding

**Fig. 4:**
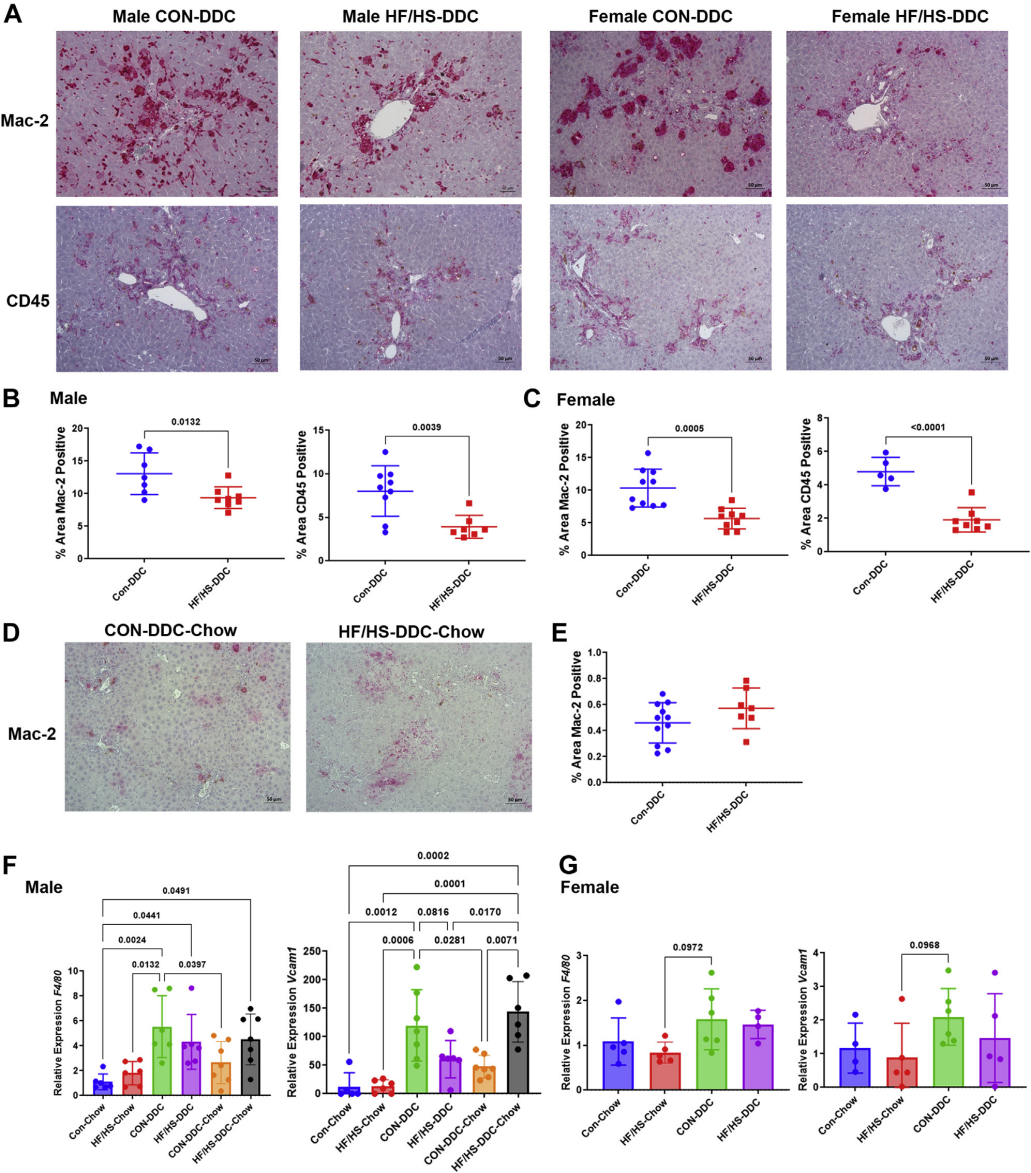
DDC feeding blunts inflammation in offspring exposed to maternal obesogenic diet. A: Representative photomicrographs of IHC for Mac-2 and CD45 in male and female offspring fed DDC. B: Quantification of Mac-2 and CD45 staining from male offspring fed DDC. C: Quantification of Mac-2 and CD45 staining from female offspring fed DDC. D: Representative photomicrographs of IHC for Mac-2 in male offspring from recovery cohort. E: Quantification of Mac-2 staining in male offspring from recovery cohort. F: Gene expression analysis by qPCR for *F4/80* and *Vcam1* in male offspring fed DDC. Recovery cohort data included. G: Gene expression analysis by qPCR for *F4/80* and *Vcam1* in female offspring fed DDC. Quantitative data presented as mean ± SD with n ≥ 5 in each group and ≥5 separate litters represented in each group. *P* values as indicated on graph. DDC, 3,5-diethoxycarbonyl-1,4-dihydrocollidine; HF/HS, high fat/high sucrose.

**Fig. 5:**
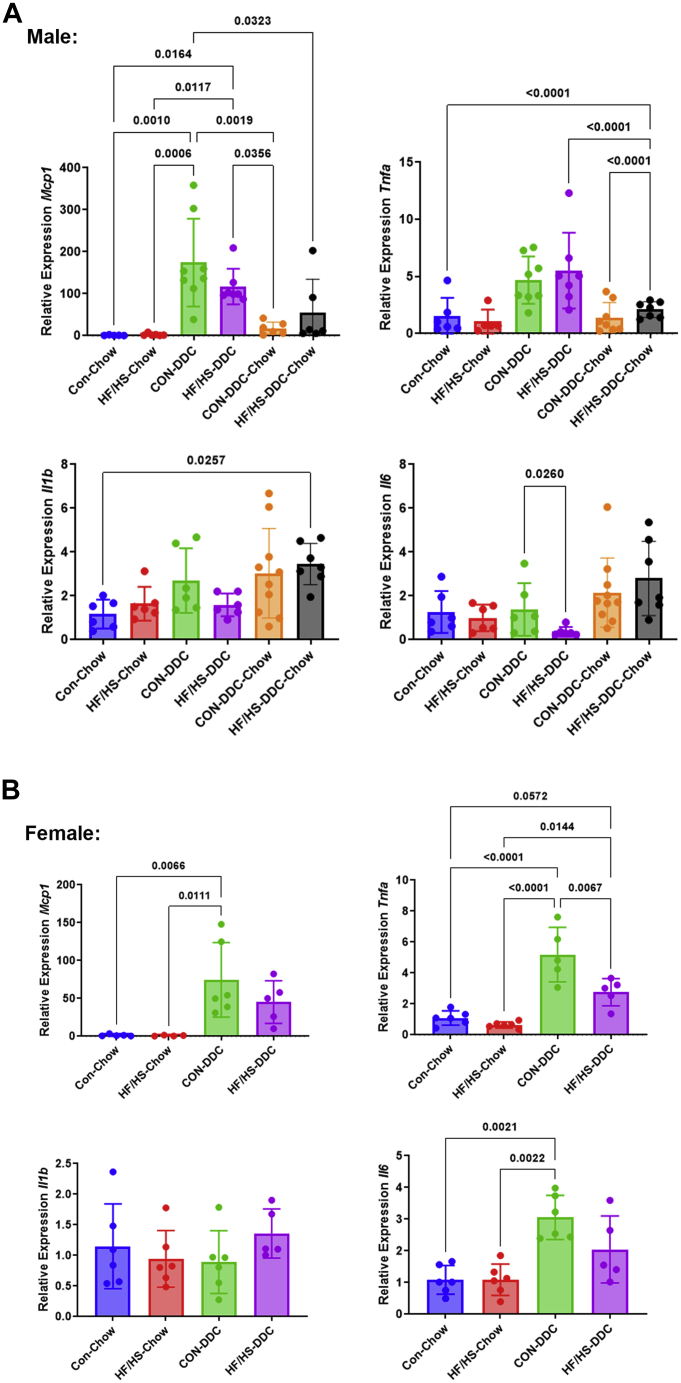
Cytokine expression after DDC feeding in offspring. A: Relative expression of proinflammatory cytokine genes in male offspring at baseline, after 2 weeks of DDC, and 2 weeks of DDC with 10 days of chow diet for recovery. B: Relative expression of proinflammatory cytokine genes in female offspring at baseline and after 2 weeks of DDC. Quantitative data presented as mean ± SD with n ≥ 5 in each group and ≥5 separate litters represented in each group. *P* values as indicated on graph. DDC, 3,5-diethoxycarbonyl-1,4-dihydrocollidine; HF/HS, high fat/high sucrose.

To evaluate the extent of liver injury due to DDC feeding, inflammation and fibrosis were assessed in each group. To assess inflammation, we undertook immunohistochemical staining for Mac-2 (macrophages) and CD45 (monocytes) (Fig. 4A, B). We observed decreased percent area Mac-2 and CD45 positive in both male and female HF/HS offspring compared to CON following DDC feeding (Fig. 4B, C). No differences in *F4/80* and *Vcam1* gene expression were observed between HF/HS and CON offspring (Fig. 4F, G). We evaluated gene expression of several inflammatory cytokines (*Mcp1*, *Tnfa*, *Il1b*, and *Il6*) in male and female offspring (Fig. 5A, B). Only expression of *Il6* was decreased in male HF/HS offspring fed DDC compared to CON (Fig. 5A).

**Fig. 6:**
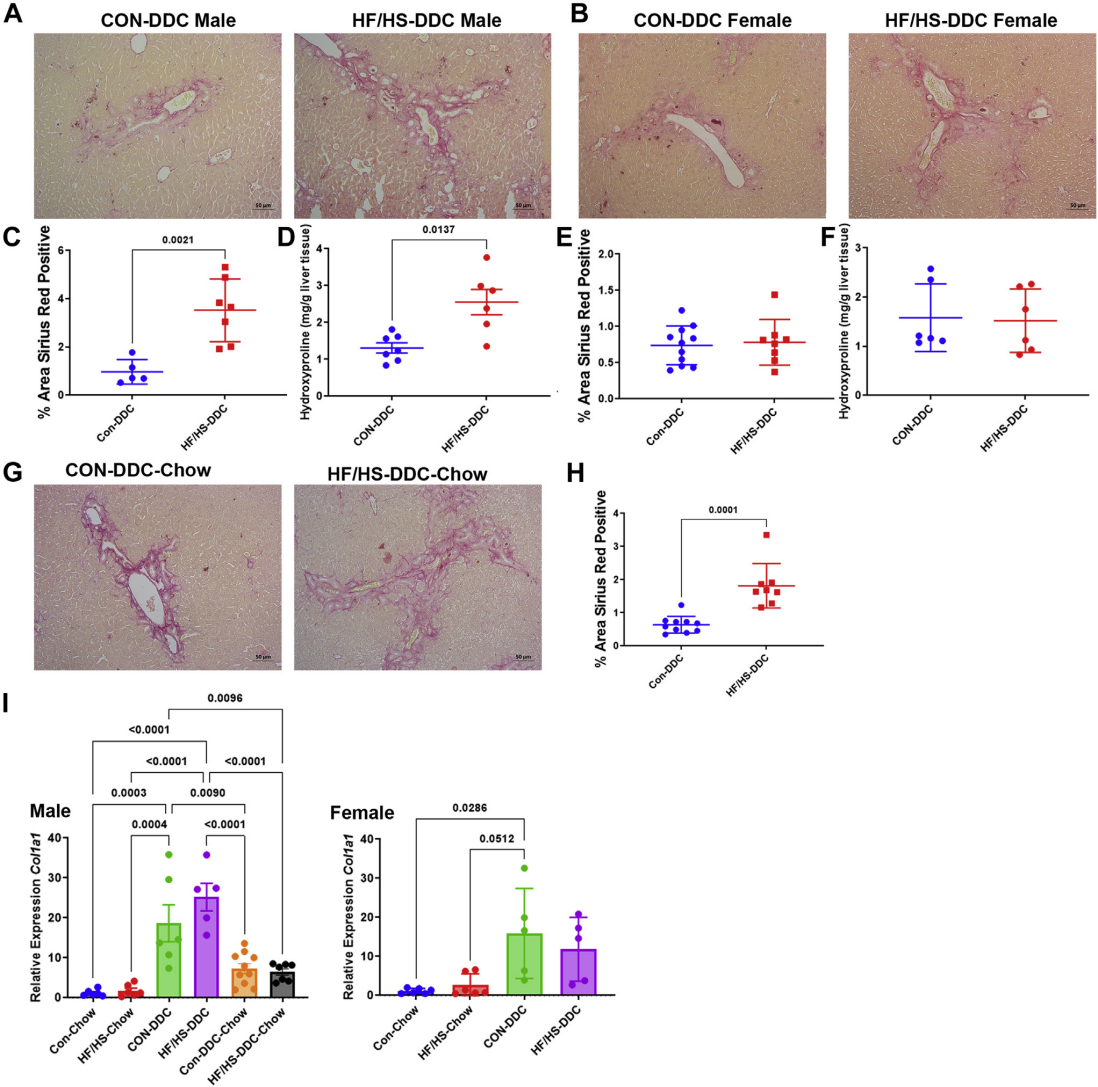
Increased fibrosis after DDC feeding with delayed resolution in male offspring exposed to maternal obesogenic diet. A: Representative photomicrographs of PSR staining of liver from male offspring fed DDC. B: Representative photomicrographs of PSR staining of liver from female offspring fed DDC. C: Percent area of PSR staining in liver of male offspring fed DDC. D: Hepatic hydroxyproline content in male offspring fed DDC. E: Percent area of PSR staining in liver of female offspring fed DDC. F: Hepatic hydroxyproline content in female offspring fed DDC. G: Representative photomicrographs of PSR staining of liver from male offspring fed DDC followed by chow for recovery. H: Percent area of PSR staining in liver of male offspring during recovery. Gene expression analysis by qPCR for *Col1a1* in liver of male offspring fed DDC. I: Gene expression analysis by qPCR for *Col1a1* in liver of female offspring fed DDC. Quantitative data presented as mean ± SD with n ≥ 5 in each group and ≥5 separate litters represented in each group. *P* values as indicated on graph. DDC, 3,5-diethoxycarbonyl-1,4-dihydrocollidine; HF/HS, high fat/high sucrose; PSR, picrosirius red.

Despite some histologic evidence of less macrophage and monocyte infiltration, offspring exposed to maternal obesogenic diet developed more extensive fibrosis. PSR staining showed evidence of periductal fibrosis in both male and female offspring fed DDC (Fig. 6A, B). Percent area PSR positive and hydroxyproline content was increased in male HF/HS offspring after 2 weeks of DDC feeding (Fig. 6C, D). No difference in *Col1a1* mRNA abundance was present in male HF/HS offspring fed DDC compared to CON (Fig. 6I). No difference in percent area PSR positive, hydroxyproline content, or *Col1a1* mRNA abundance was observed between HF/HS and CON female offspring fed DDC (Fig. 6E, F, I).

**Fig. 7:**
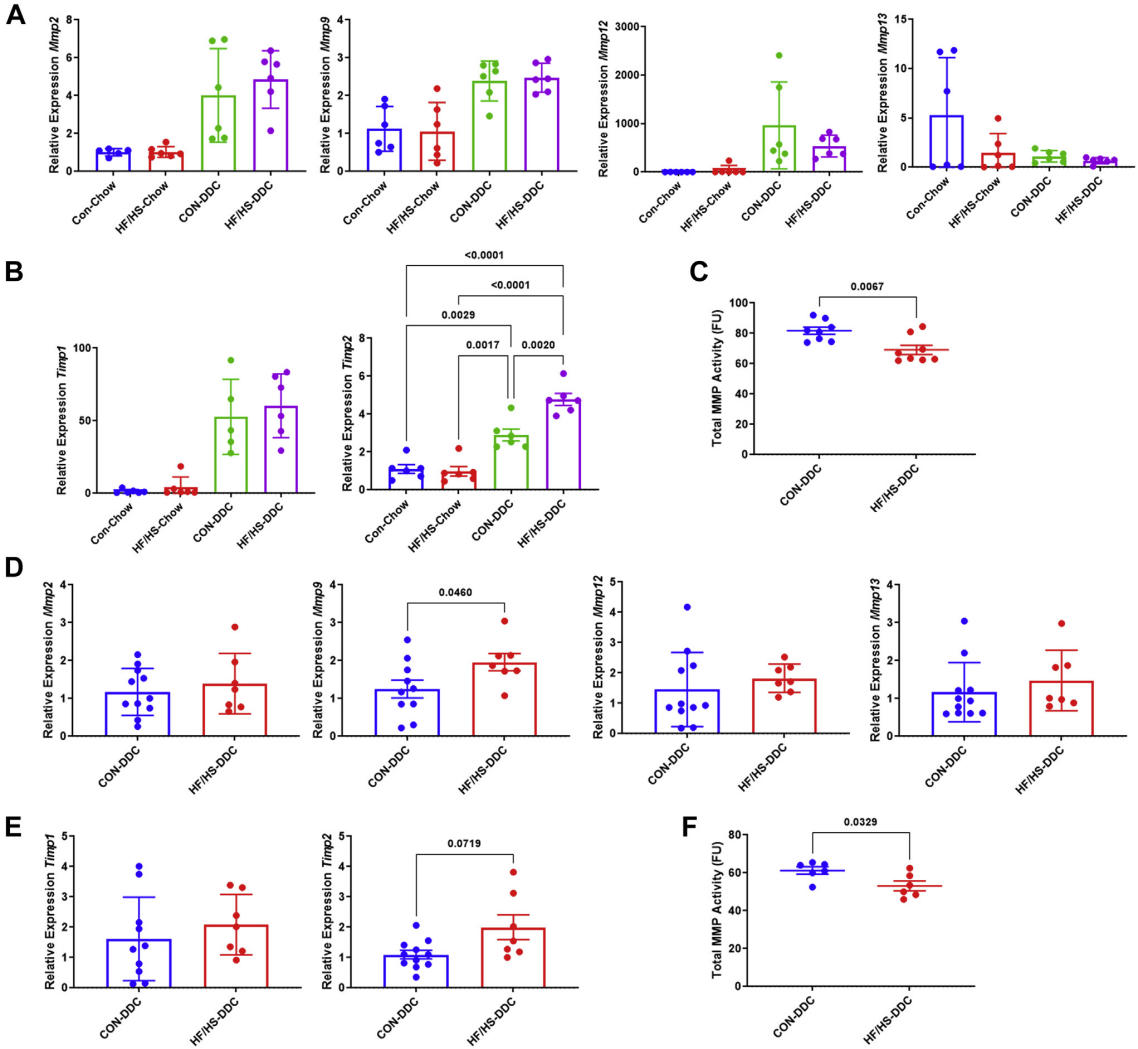
Decreased MMP activity in liver of offspring exposed to maternal obesogenic diet during DDC feeding and recovery. A: Gene expression analysis by qPCR for *Mmp2*, *Mmp9*, *Mmp12*, and *Mmp13* in livers of male offspring fed DDC. B: Gene expression analysis by qPCR for *Timp1* and *Timp2* in livers of male offspring fed DDC. C: MMP enzymatic activity in livers of male offspring fed DDC. D: Gene expression analysis by qPCR for *Mmp2*, *Mmp9*, *Mmp12*, and *Mmp13* in liver of male offspring fed DDC followed by chow for recovery. E: Gene expression analysis by qPCR for *Timp1* and *Timp2* in liver of male offspring fed DDC followed by chow for recovery. F: MMP enzymatic activity in liver of male offspring fed DDC followed by chow for recovery. Quantitative data presented as mean ± SD with n ≥ 5 in each group and ≥5 separate litters represented in each group. *P* values as indicated on graph. DDC, 3,5-diethoxycarbonyl-1,4-dihydrocollidine; HF/HS, high fat/high sucrose.

To better understand the mechanisms underlying the increased fibrosis in offspring from the maternal HF/HS lineage, we measured MMP and TIMP gene expression. Expression of *Mmp2*, *Mmp9*, *Mmp12*, *Mmp13* (Fig. 7A), and *Timp1* were unchanged, while *Timp2* expression increased (Fig. 7B). Total MMP activity was decreased in HF/HS offspring compared to CON offspring fed DDC (Fig. 7C). These data suggest that the increased fibrosis may in part reflect altered tissue remodeling.

### Delayed recovery from cholestatic liver injury in offspring exposed to maternal obesogenic diet

To assess the ability of maternal HF/HS lineage offspring to resolve cholestatic injury, mice in both groups were switched back to chow diet after 2 weeks of DDC. Male offspring were selected because of the greater differences in fibrosis observed after 2 weeks of DDC diet (Fig. 6A). At 10 days after reversal to chow, we found no difference in body or liver weight or liver weight to body weight ratio between groups (Fig. 2A–D). However, we observed sustained ductular reaction in maternal HF/HS lineage offspring as evidenced by increased density of CK-19-positive ducts and *Krt19* expression by qPCR (Fig. 3C, D). Mac-2 staining was not statistically different between groups during the recovery phase, although *Vcam1* and *Tfna* showed increased expression (Figs. 3D–F and 4A). Other inflammatory cytokine expression was unchanged between CON and HF/HS offspring during the recovery period. PSR staining showed greater collagen deposition in maternal HF/HS offspring compared to CON (Fig. 5G, H).

We next asked if delayed resolution of injury is associated with changes in MMP activity by measuring expression of MMP and TIMP transcripts after 10 days of recovery. Expression of *Mmp2*, *Mmp12*, and *Mmp13* were unchanged (Fig. 7D), while expression of *Mmp9* in liver of offspring from the maternal HF/HS lineage increased (Fig. 7D). Expression of *Timp1* was similar between groups, but expression of *Timp2* was again increased in offspring from the maternal HF/HS lineage (Fig. 7E). As observed 2 weeks after DDC feeding, total MMP activity was reduced in offspring from maternal HF/HS during recovery (Fig. 7F). These data again support that increased fibrosis may in part reflect altered tissue remodeling.

### No difference in BA homeostasis in maternal obesogenic diet fed offspring during DDC feeding

**Fig. 8:**
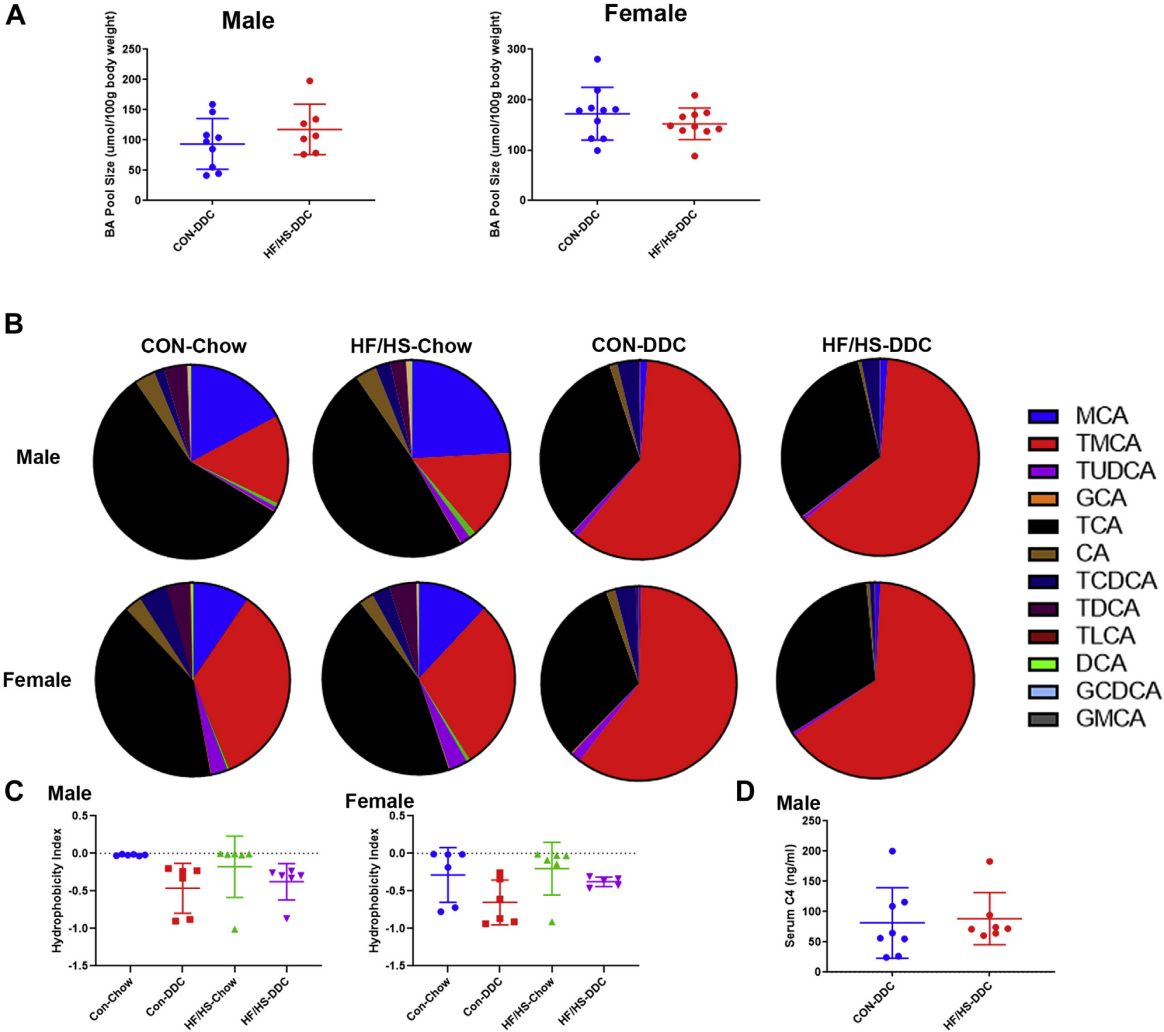
No difference in BA homeostasis after DDC feeding in maternal CON and HF/HS offspring. A: BA pool size male and female offspring fed DDC. B: Abundance of BA species in livers of male and female offspring fed DDC. C: Hydrophobicity index for liver BAs in male and female offspring at baseline and after 2 weeks DDC feeding. D: Serum C4 concentrations in male offspring fed DDC. Quantitative data presented as mean ± SD with n ≥ 5 in each group and ≥5 separate litters represented in each group. *P* values as indicated on graph. BA, bile acid; DDC, 3,5-diethoxycarbonyl-1,4-dihydrocollidine; HF/HS, high fat/high sucrose.

**Fig. 9:**
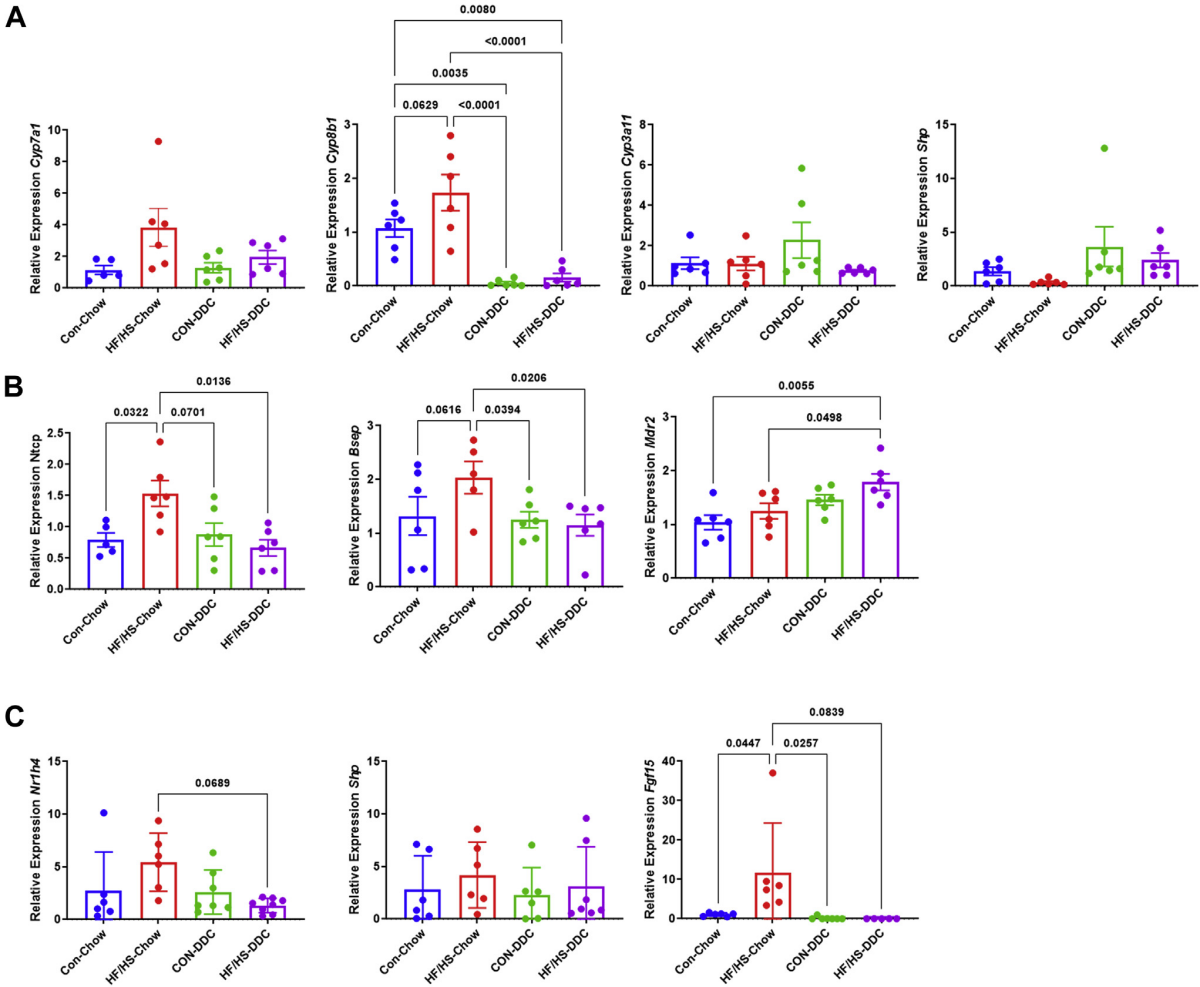
Expression of genes involved in BA metabolism after DDC feeding in maternal CON and HF/HS offspring. A: Gene expression analysis by qPCR for *Cyp7a1*, *Cyp8b1*, *Cyp3a11*, and *Shp* in liver of male offspring. B: Gene expression analysis by qPCR for *Bsep*, *Ntcp*, and *Mdr2* in liver of male offspring. C: Gene expression analysis by qPCR for *Nr1h4*, *Shp*, and *Fgf15* in distal ileum of male offspring. Quantitative data presented as mean ± SD with n ≥ 5 in each group and ≥5 separate litters represented in each group. *P* values as indicated on graph. BA, bile acid; DDC, 3,5-diethoxycarbonyl-1,4-dihydrocollidine; HF/HS, high fat/high sucrose.

We previously reported that maternal obesogenic diet exposure alters BA homeostasis in offspring with an increase in BA pool size and a shift in intrahepatic BA species (17). To assess overall BA homeostasis during cholestasis, BA pool size was measured after 2 weeks of DDC feeding. No difference in pool size was observed between male or female HF/HS lineage offspring compared to CON after DDC feeding (Fig. 8A). Relative abundance of specific intrahepatic BA species demonstrated a shift to primarily tauromuricholic acid in all groups without a clear difference between groups after DDC feeding (Fig. 8B). There was also no significant difference in the BA hydrophobicity index and serum C4 between groups after DDC feeding (Fig. 8C, D). After DDC feeding, we found no difference in *Cyp7a1*, *Cyp8b1*, *Cyp3a11*, and *Shp* mRNA abundances (Fig. 9A). There were no differences in relative abundance of BA transporter transcripts *Bsep*, *Ntcp*, and *Mdr2* after DDC feeding (Fig. 9B). There were also no significant differences in ileal expression of *Nr1h4*, *Shp*, or *Fgf15* between HF/HS and CON offspring after DDC feeding (Fig. 9C). These data suggest that fibrotic injury in DDC fed mice is not associated with alterations in either BA pool size or composition or changes in BA synthesis.

### Microbiome and cholestatic liver injury in offspring exposed to maternal obesogenic diet

**Fig. 10:**
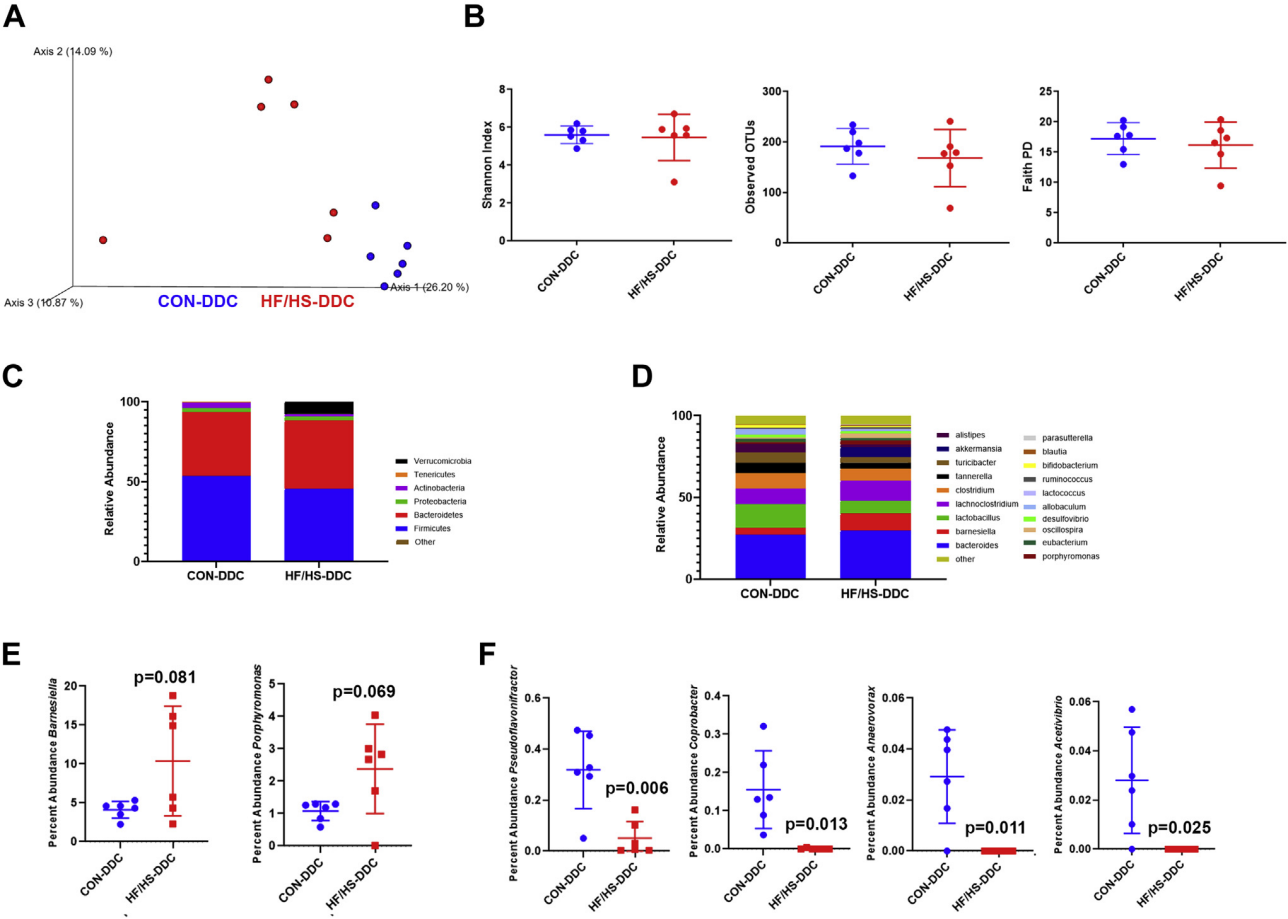
Shift in the cecal microbiome of HF/HS offspring fed DDC. A: Bray-Curtis plots for beta-diversity of cecal microbiome from HF/HS and CON offspring. B: Measures of alpha-diversity (Observed OTUs, Faith PD, and Shannon Diversity) in cecal microbiome of HF/HS and CON offspring. C: Relative abundance of each bacterial phyla in cecal microbiome of HF/HS and CON offspring. D: Relative abundance of each bacterial genus in cecal microbiome of HF/HS and CON offspring. E: High abundance genera with a trend toward a difference in cecal microbiome of HF/HS and CON offspring. F: Genera with significantly differential abundance in cecal microbiome of HF/HS and CON offspring. Quantitative data presented as mean ± SD with n = 6 in each group and ≥5 separate litters represented in each group. *P* values as indicated on graph. DDC, 3,5-diethoxycarbonyl-1,4-dihydrocollidine; HF/HS, high fat/high sucrose.

We considered another potential mechanism for passage of phenotypes from one generation to the next, namely through the microbiome. An altered offspring microbiome has been described in the setting of maternal obesogenic diet exposure and is a potential mechanism for enhanced cholestatic liver injury observed in the offspring (22, 23). We previously reported shifts in the offspring microbiome after maternal HF/HS exposure (24). Principal component analysis of microbial 16S sequences demonstrated that CON and HF/HS offspring fed DDC diverged in community content, though this was not statistically significant (*P* = 0.07) (Fig. 10A). No difference was observed between the two groups in several measures of alpha diversity (Fig. 10B). At the family level, only Tenericutes showed a statistically significant difference between groups (Fig. 10C, D), though at the genus level, there were several differential abundances (Fig. 10C). Among higher abundance genera, *Barnesiella* and *Porphyromonas* show a trend toward increase in HF/HS offspring (Fig. 10E). Genera in which we found statistically significant differences include *Pseudoflavonifractor*, *Coprobacter*, *Anaerovorax*, and *Acetivibrio* (Fig. 10F). Other genera showing a trend toward a difference include *Paraeggerthella*, *Robinsoniella*, *Paludibacter*, and *Holdemania* (Fig. 10G).

**Fig. 11:**
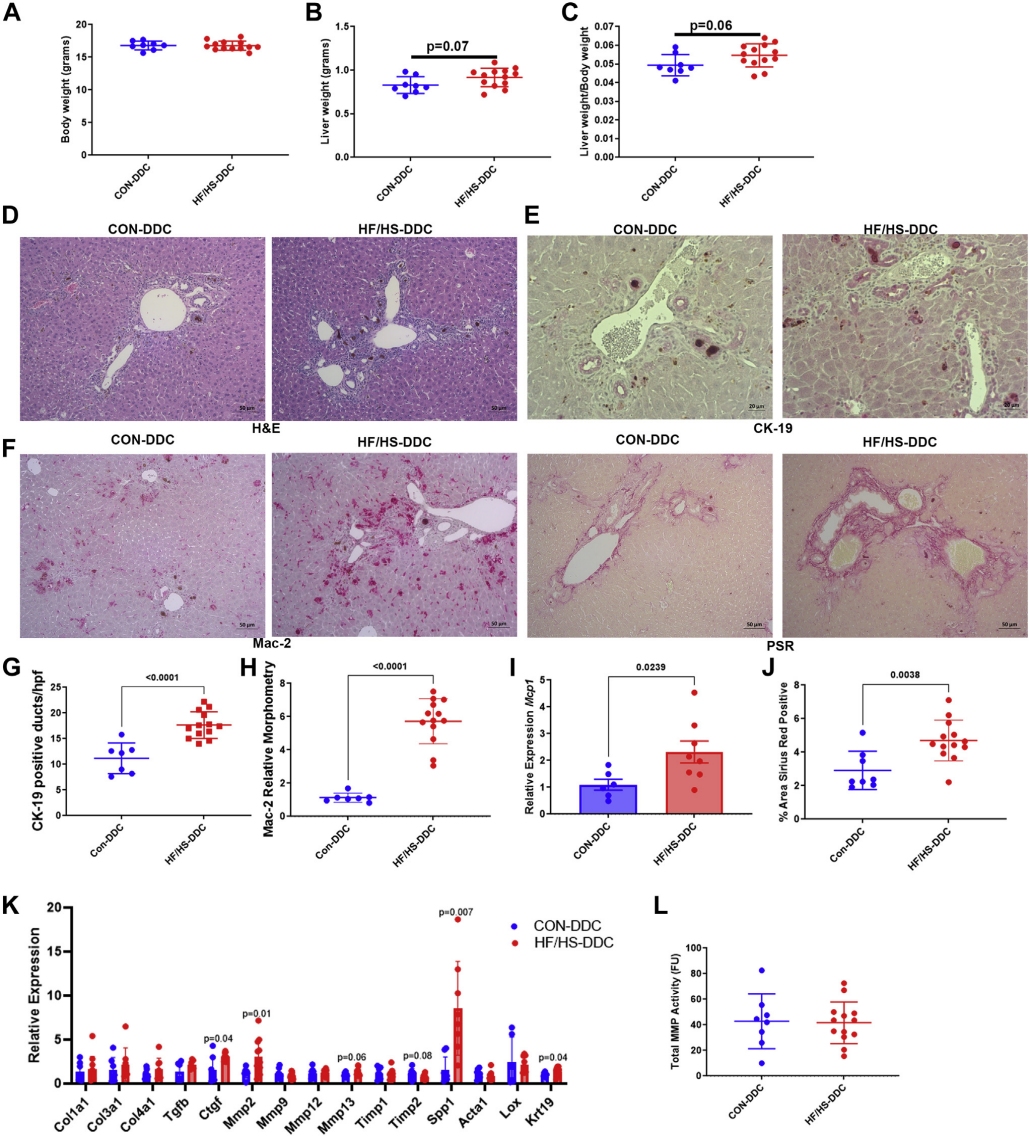
Transfer of the cecal microbiome from HF/HS lineage offspring leads to worse cholestatic liver injury. A: BW of CMT recipient mice fed DDC. B: LW of recipient mice fed DDC. C: LW/BW of recipient mice fed DDC. D: Representative photomicrographs of H&E staining of liver from CMT recipient mice fed DDC. E: Representative photomicrographs of IHC for CK-19 in liver of CMT recipient mice fed DDC. F: Quantification of CK-19-positive bile ducts and gene expression analysis by qPCR for *Krt19* in livers of CMT recipient mice fed DDC. G: Representative photomicrographs of Mac-2 IHC and PSR staining in liver of CMT recipient mice fed DDC. H: Quantification of Mac-2 staining in livers of CMT recipient mice fed DDC. I: Gene expression analysis by qPCR for *Mcp1* in liver of CMT recipient mice fed DDC. J: Quantification of PSR staining in livers of CMT recipient mice fed DDC. K: Gene expression analysis by qPCR for *Col1a1*, *Mmp2*, and *Mmp13* in livers of CMT recipient mice fed DDC. L: MMP enzymatic activity in livers of CMT recipient mice fed DDC. Donor and recipient mice were male. Quantitative data presented as mean ± SD with n ≥ 6 in each group. *P* values as indicated on graph. CMT, cecal microbiome transplantation; DDC, 3,5-diethoxycarbonyl-1,4-dihydrocollidine; HF/HS, high fat/high sucrose; LW/BW, liver to body weight.

To evaluate whether changes in the microbiome may promote the enhanced response to DDC exposure, we performed cecal microbiome transplantation (CMT) from male CON or HF/HS offspring to antibiotic-treated male mice followed by 2 weeks of DDC exposure. After 2 weeks of DDC feeding, body weights did not differ between CON and HF/HS recipient mice (Fig. 11A). There was a trend toward increased liver weight (*P* = 0.07) and Liver to body weight (LW/BW) ratio (*P* = 0.06) (Fig. 11B, C) and ductular reaction was increased in HF/HS recipient mice after DDC feeding (Fig. 11D, E). Macrophage expansion and expression of *Mcp1* was increased in HF/HS recipient mice (Fig. 11F, H, I). Fibrosis as indicated by PSR staining was increased in HF/HS recipient mice after DDC feeding (Fig. 11F, J). We measured a panel of RNAs associated with fibrosis with a significant increase observed in *Ctgf*, *Spp1*, and *Mmp2* in HF/HS recipients (Fig. 11K). There was a trend toward an increase in *Timp2* and *Mmp13* in HF/HS recipients (Fig. 11K). No difference was present in MMP activity (Fig. 11L). These data suggest that the increased fibrosis found following CMT from male HF/HS offspring reflects increased inflammatory signaling and increased expression of factors involved in fibrosis.

**Fig. 12:**
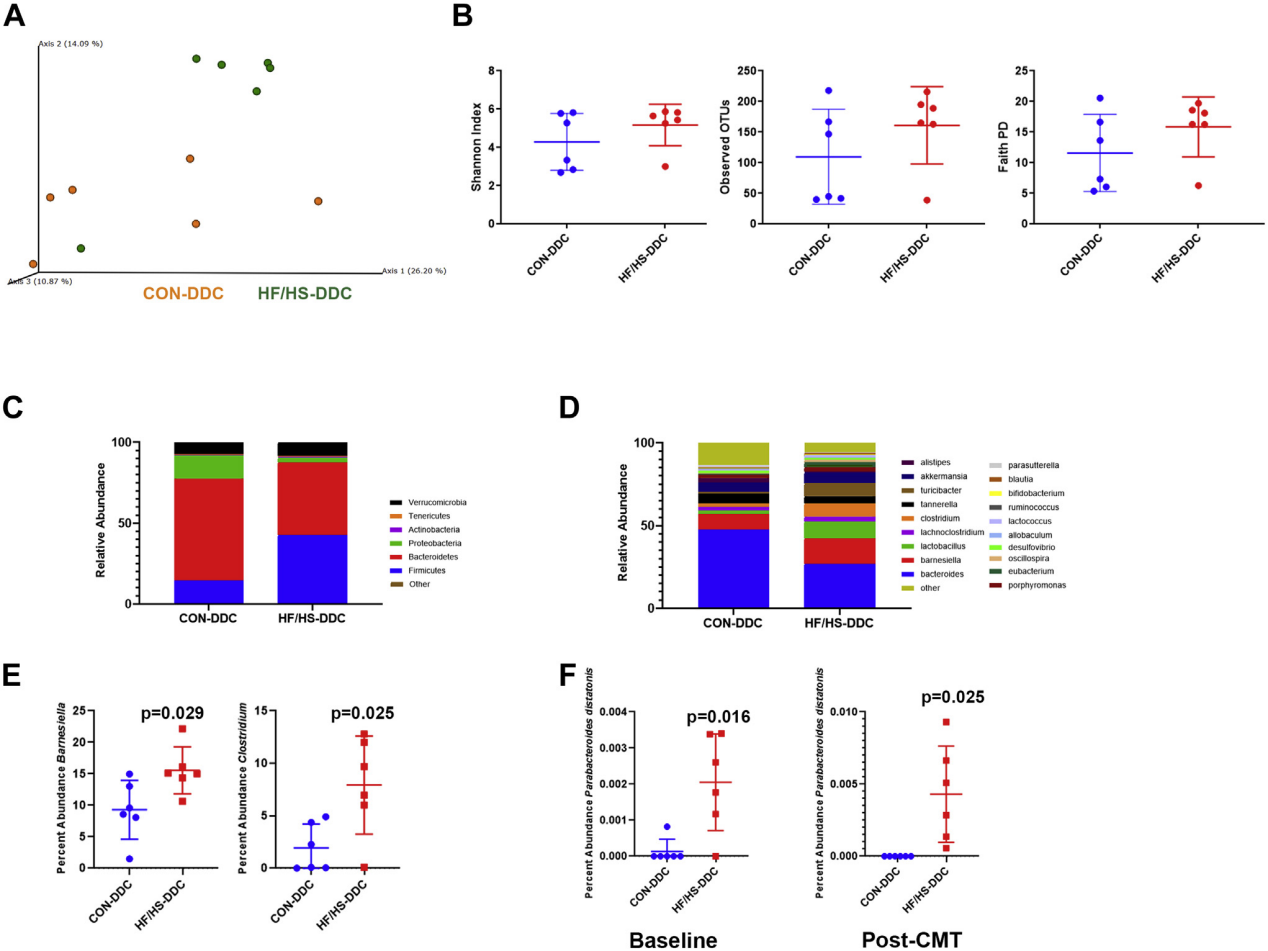
Shift in the cecal microbiome of HF/HS CMT recipient mice fed DDC. A: Bray-Curtis plots for beta-diversity of cecal microbiome from HF/HS and CON CMT recipients. B: Measures of alpha-diversity (Observed OTUs, Faith PD, and Shannon Diversity) in cecal microbiome of HF/HS and CON CMT recipients. C: Relative abundance of each bacterial phyla in cecal microbiome of HF/HS and CON CMT recipients. D: Relative abundance of each bacterial genus in cecal microbiome of HF/HS and CON CMT recipients. E: Genera with significantly differential abundance in cecal microbiome of HF/HS and CON CMT recipients. F: Abundance of *Parabacteroides distasonis* in baseline offspring fed DDC and CMT recipient mice fed DDC. Donor and recipient mice were male. Quantitative data presented as mean ± SD with n = 6 in each group and ≥5 separate litters represented in each group. *P* values as indicated on graph. CMT, cecal microbiome transplantation; DDC, 3,5-diethoxycarbonyl-1,4-dihydrocollidine; HF/HS, high fat/high sucrose.

We further evaluated the gut microbiome in CMT recipient mice after 2 weeks of DDC feeding by 16S sequencing. Principal component analysis demonstrated that bacterial populations from CON and HF/HS recipient mice were distinct (*P* = 0.04) (Fig. 12A). There was no statistically significant difference in measures of alpha-diversity (Fig. 12B). At the phylum level, there was an increase in *Firmicutes* and a decrease in *Bacteroidetes* abundance in HF/HS recipient offspring after DDC feeding. There was also a decrease in the abundance of *Proteobacteria* (Fig. 12C). At the genus level, there was a statistically significant increase in *Barnesiella* and *Clostridium* (Fig. 12D, E). We identified *Parabacteroides distasonis* as a species increased in HF/HS recipient mice (Fig. 12F); this species was also increased in HF/HS offspring fed DDC for 2 weeks.

## Discussion

Our work confirms that maternal HF/HS offspring exhibit enhanced ductular reaction and fibrotic response to DDC with delayed recovery from this injury. Worsened cholestatic liver injury is also transmissible, supporting the concept that an altered microbiome might be causal. To our knowledge, this is the first demonstration of a link between maternal diet and cholestatic disease in offspring. Our data have multiple implications.

### Maternal obesogenic diet enhances ductular reaction and fibrosis during cholestatic liver injury

Ductular reaction or proliferation is commonly described in cholestatic liver diseases and consistently associated with the development of fibrosis (25). In our model of maternal HF/HS diet exposure, offspring show periportal injury with mild periductal inflammation and fibrosis. The current findings likely reflect exacerbation of underlying periportal injury after mice are fed a cholestasis-inducing diet. There is also evidence of impaired remodeling with a decrease in MMP activity at 2 weeks after DDC feeding and during the recovery phase. This was associated with an increase in expression of *Timp2*.

One potential etiology is periportal injury driven by an altered microbiome. Transfer of the microbiome from HF/HS offspring to microbiome-depleted mice is sufficient to increase cholestatic liver injury. HF/HS recipient mice developed more ductular reaction, inflammation, and fibrosis during DDC feeding. We did not observe the same decrease in MMP activity although *Mmp2* expression was increased and is reported to be increased and have a profibrogenic role in liver fibrosis (26). We also observed an increase in other factors associated with fibrosis including connective tissue growth factor and osteopontin (27, 28). Future studies will identify which factors are essential in developmental programming of cholestasis-induced liver fibrosis after maternal obesogenic diet exposure.

Another consideration is that the altered microbiome may impair intestinal barrier and increase translocation of bacterial products into the portal circulation (29). Indeed, stool transplantation from infants of obese mothers to gnotobiotic mice induces portal inflammation and alters gut barrier function as evidenced by FITC-dextran translocation and decreased intestinal *Tjp1* expression (22). Also, offspring exposed to maternal HFDs have impaired gut barriers and are more sensitive to dextran sodium sulfate colitis (15). Impaired intestinal barrier function in offspring of obese mothers could enable entry of lipopolysaccharide and other bacterial products into the portal circulation which could prime periportal injury. Our studies support a role for maternal diet–induced changes in the microbiome in increase susceptibility to cholestatic liver injury.

### Recovery from injury is delayed in offspring exposed to maternal obesogenic diet

The liver is capable of hepatobiliary repair following cholestatic liver injury via cholangioctye proliferation and remodeling of damaged hepatic tissue. This process may also include transdifferentiation of hepatocytes (30). We found that while offspring of the maternal CON lineage repaired periportal injury, offspring of the maternal HF/HS lineage sustained ductular reaction, periportal inflammation, and fibrosis. Despite the continued periportal injury, the HF/HS and CON lineage had similar weight gain. One possible explanation for this finding is that the hepatobiliary repair processes are blunted in offspring exposed to maternal HF/HS diet, but further studies will be required to resolve the underlying mechanisms dissociating weight gain and resolution of cholestatic injury.

### BA perturbations normalize during DDC feeding

We previously reported that BA homeostasis is altered in offspring exposed to maternal HF/HS diet (17). Notably, BA pool size is increased, and there is a shift in the abundance of specific intrahepatic BA species. The failure of DDC to change BA pool size in male or female offspring could reflect decreased BA production after DDC feeding, evidenced by decreased *Cyp7a1* and *Cyp8b1* expression (31). At baseline, offspring from the maternal HF/HS lineage exhibit increased *Cyp7a1* expression and activity compared to CON, a difference that was lost after DDC feeding. We also note that differences in intrahepatic BA profiles in maternal HF/HS lineage offspring at baseline were lost after DDC feeding as both groups exhibit an intrahepatic BA pool dominated by muricholic acid species. Given these findings, shifts in BA metabolism are unlikely to be involved in the increased hepatobiliary injury in offspring exposed to maternal obesogenic diet.

### Shifts in offspring microbiome after maternal obesogenic diet exposure drives increased cholestatic liver injury

One mode of transmission of developmentally programmed phenotypes is for a dam to confer an altered microbiome to offspring, and we demonstrate that the gut microbiome in HF/HS offspring is sufficient to worsen cholestatic liver injury. The increased *Firmicutes* to *Bacteroidetes* ratio in the phenotype-transmitting microbial community is also observed in HF/HS offspring at baseline (unpublished data). We identified *Barnesiella* among the genera significantly increased in HF/HS recipients after DDC feeding. Intriguingly, gut bacterial populations in PSC patients with IBD are also enriched in *Barnesiella* (32) compared to IBD patients without PSC. We also observed increased relative abundances in *Parabacteroides* in HF/HS offspring and HF/HS CMT recipients fed DDC. Studies of *Parabacteroides* in patients with PSC have produced conflicting results with levels being increased or decreased in PSC patients (33, 34). Future studies evaluating specific taxa will be necessary to confirm taxa-specific drivers of cholestatic liver disease in people and mice to discern mechanisms.

### Sex differences in the maternal obesogenic diet programming of cholestatic liver disease

Understanding factors that affect sex differences in cholestatic liver disease is important as differences in prevalence and disease progression have been reported. Notably PSC is more common in men at an approximate ratio of 2:1 (35). Women also had a greater period of transplant-free survival (36). In our model, we observe a similar increase in male and female HF/HS offspring ductular reaction after DDC diet feeding. However, development of fibrosis was only increased in male offspring. While the mechanism for this sex difference was not explored in this study, prior work has noted sex differences in hepatic pathology after maternal obesogenic diet exposure. Maternal HFD programming of hepatic steatosis is worse in male offspring in association with differences in shifts in the offspring microbiome (37). Maternal obesogenic diet exposure also has sex-dependent effects on the offspring hepatic transcriptome suggesting that there are differences in epigenetic modifications induced by the maternal exposure (38). Future studies will need to focus on the potential mechanism of these sex differences identified in preclinical models and whether they provide an explanation for sex differences observed in clinical practice.

Several limitations of this study warrant further discussion. As just noted, there are some sex differences that exist in disease progression in our model. While our study was able to identify pathologic differences, it was not designed to identify mechanistic differences between sex. This will be an essential area for further evaluation given the previously noted sex differences in PSC (35, 36). A global analysis of differences in microbiome and epigenetic modifications induced by maternal diet will likely provide an answer to this important question. Another limitation of the current study is that it does not address all potential mechanisms of developmental programming. While the microbiome is an important factor, there are likely epigenetic modifications playing a role in the observed phenotype in our model. This is highlighted by the discrepancy in inflammation observed in this study with HF/HS offspring showing less inflammation after DDC diet feeding, while recipients of cecal contents from HF/HS offspring develop worse inflammation. This supports that there are other modifiers of the inflammatory response than the maternal diet–induced changes in the offspring microbiome. Further work in our lab will focus on identifying the factors behind this discrepancy as well as the mechanism by which fibrosis is worse in HF/HS offspring despite less inflammation.

In summary, maternal HF/HS diet exposure increases ductular reaction and fibrosis during DDC-induced cholestatic liver injury. Offspring exposed to maternal HF/HS diet delays hepatobiliary repair response. The mechanism for these changes is at least partly mediated by an altered microbiome in HF/HS lineage offspring. We propose that the correlation of maternal diet and development of cholestatic liver diseases in humans could be a fruitful line of investigation.

## Data availability

All data are contained within the manuscript. Data requests can be made to Michael Thompson, Washington University School of Medicine, at thompsonmd@wustl.edu.

## Conflict of interest

The authors declare that they have no conflicts of interest with the contents of this article.
